# Patient Perceptions on the Virtual Amyotrophic Lateral Sclerosis Clinic during COVID-19

**DOI:** 10.1017/cjn.2021.160

**Published:** 2021-07-13

**Authors:** Sophy Chan-Nguyen, Mustafa Karacam, Benjamin Ritsma, Ramana Appireddy

**Affiliations:** Department of Family Medicine, Queen’s University, Kingston, Ontario, Canada; Division of Neurology, Department of Medicine, Queen’s University, Kingston, Ontario, Canada; School of Kinesiology and Health Sciences, Queen’s University, Kingston, Ontario, Canada; Department of Physical Medicine and Rehabilitation, Queen’s University, Kingston, Ontario, Canada

**Keywords:** Amyotrophic lateral sclerosis, quality of care

Patients with amyotrophic lateral sclerosis (ALS) and their caregivers often attend specialized multidisciplinary ALS clinics, where multiple physicians and allied healthcare professionals deliver and coordinate care.^1^ The management of patients in such a multidisciplinary clinic (MDC) is considered as one of the best practice recommendations for ALS care in Canada.^2^ There is a recognized burden of fatigue, travel, out-of-pocket expenses, and time to attend in-person outpatient care, which is greater for ALS patients.^3^ Virtual care (VC) has the potential to address some of this burden. VC is defined as any remote interaction between patients and healthcare providers using any form of communication or information technology (e.g. telephone, video conference, email, or text messaging) to deliver care.^4^ In line with the widespread adoption of VC in Canada during the COVID-19 pandemic, the ALS MDC (affiliated with Queen’s University in Kingston, Ontario) transitioned to broader utilization of VC.^5^ Recent literature suggests the feasibility of VC in ALS clinics, along with potential cost savings and better patient experience.^3^ This paper explores the benefits and challenges of utilizing virtual MDCs for ongoing ALS care and contributes to ongoing research exploring accessible ALS care in and beyond the pandemic.

We used a case study research design to provide a generalizable, but also a complex portrayal of patient and caregiver experiences of in-person and virtual MDCs.^6^ In a collaboration between clinicians at Providence Care Hospital and Kingston Health Sciences Centre, the Kingston ALS MDC (in-person and virtual) is undertaken at one site by a team of healthcare professionals from Neurology, Respirology, Physical Medicine & Rehabilitation/Physiatry, Palliative Care, Respiratory Therapy, Physiotherapy, Occupational Therapy, Speech–Language Pathology, and nursing. ALS patients who completed at least one virtual MDC between March and August 2020 were approached regarding interest in study participation. If the patient could not participate in the interview, we invited a caregiver who had experienced a virtual ALS MDC with the patient to participate. This study is approved by the Queen’s University’s Health Sciences and Affiliated Teaching Hospitals Research Ethics Board.

Between March 2020 and August 2020, two study team members conducted semi-structured interviews using an audio recorder over the phone. Based on initial interest, we invited 20 patients to participate. Thirteen individuals initially wished to participate. However, 1 withdrew and 1 participant expired before data collection. Eleven individuals (7 patients and 4 caregivers) participated in the study. A hired transcriptionist transcribed 11 interviews verbatim. The study team also collected the medical and sociodemographic profiles of each participant. Two study team members analyzed the 11 transcripts iteratively using NVivo 10 software. We used a thematic analysis approach to examine patient and caregiver experiences of virtual MDC. Two team members generated initial codes and themes. After coding every third interview, the research team reviewed the initial codes and took notes on major recurring themes. Following initial coding, the researchers convened to review and establish main themes and sub-themes. All sociodemographic and medical details of the patients are presented in Table 1.

**Table 1:** Sociodemographic profile of the participants and the frequency of in-person and virtual care visits

Patient	Age	Sex	Location of residence	Caregiver assistance required	One-way distance and travel time from home to hospital(KM/min)	Type of virtual care	ALS diagnosis (years)	ALS onset (location)	Mobility status	Degree of disability/Other ADLs	Non-invasive ventilation	Enteral feeding	Comorbidities
1	80	M	Rural	Yes	125 (90)	Video	5 months	Upper limb	1PA, with 4ww (short distances)	1PA	Yes	Pending	Dyslipidemia, prostate CA
2	78	M	Urban (Outside of Kingston)	Yes	46 (38)	Phone	2.5	Upper limb	Independent, no gait aid	1PA	Yes	No	Coronary artery disease
3	77	F	Urban (Outside of Kingston)	No	83 (56)	Video	4.5	Upper limb	Independent, with 4ww	1PA (minimal)	No	No	Hypertension, adhesive capsulitis, GERD, rosacea, umbilical hernia surgery,
4	64	F	Urban/Outside of Kingston	No	94 (71)	Video	6 months	Lower limb	Independent, with single point cane	Independent	No	No	Carpal tunnel syndrome, Sjogren’s disease
5	62	M	Urban (Outside of Kingston)	Yes	17 (27)	Video	9 months	Bulbar	Independent, with 4ww	Independent	No	No	Osteoarthritis, obstructive sleep apnea, Barrett’s esophagus, colonic tubular adenoma, diverticular disease, pilonidal high abscess, septoplasty, hip fracture
6	76	M	Rural	No	117 (77)	Video	3 months	Upper limb	Independent, no gait aid	1PA	No	No	Hernia, osteoarthritis, bilateral total knee replacements, left index finger partial amputation
7	64	M	Rural	Yes	39 (40)	Video	6 months	Lower limb	Independent, no gait aid	Independent	No	No	Chronic depression, dyslipidemia, left biceps tendon rupture/repair
8	77	M	Urban (Outside of Kingston)	Yes	85/72	Video	2 years	Lower limb	Independent, with 4ww	1PA	No	No	Chronic lymphocytic leukemia
9	81	M	Rural	Yes	28 (29)	Phone	8 months	Bulbar	Independent, no gait aid	Independent	Yes	Yes	HTN, dyslipidemia, cataracts, cholecystectomy, appendectomy
10	67	M	Urban (Outside of Kingston)	No	182 (113)	Video	4 years	Bulbar/ upper limb	Independent/1PA, with 4ww (short distances)	1PA (minimal)	No	No	HTN
11	69	M	Urban (Outside of Kingston)	Yes	87 (56)	Video	1.75	Lower limb	Wheelchair and 1PA with 4ww	1PA	Yes	No	Atrial fibrillation, depression

1PA= 1 person assist; ADLs = activities of daily living; 4ww = 4 wheeled walker; NIV = non-invasive ventilation.

Note: Urban (Outside of Kingston): The Kingston Health Sciences Centre has a large catchment area within southeastern Ontario that services patients who live in smaller urban areas (designated as “urban” by Statistics Canada) outside of Kingston.

Overall, participants expressed that the virtual MDC was useful as it was key to their ability to receive ongoing care during the COVID-19 pandemic. Three major themes emerged from the study (Table 2). First, the virtual clinic increased comfort and provided logistical benefits for patients by reducing travel, costs, and fatigue. Second, the virtual clinic offered ongoing ALS care in a safe manner, reducing exposure risk to COVID-19. Third, while patients identified the benefits of virtual MDCs, in-person MDCs often remained the preferred mode of ALS care for some.

**Table 2:** Summary of themes with quotes from participants

Theme	Quotes
Comfort and logistical benefits of virtual care	“I think my husband felt more comfortable during the [virtual] ALS visit because he was supposed to see five doctors, so he got to sit in one spot and wasn’t moved from room to room to room.” (P2, Caregiver) “I actually save about $250 because I had to hire somebody to take him in a special vehicle when we were going to Kingston. I can’t get him in a regular car anymore. And then, of course, again, I’m 76 years old, and all of that physical aspect is on me, right? And that was the cheapest cost.” (P10, Caregiver) “I did eventually, after two hours, get tired so I asked [physiatrist] if it would be okay if we signed off because my energy was dwindling. As soon as I clicked off I realized, hey, I’m at home, I can go lay down if I want to or I can go and relax or get something to drink or use the washroom or whatever I wanted to do. (P11, Patient)
Avoiding risk while providing care	“Hospitals are full of sick people anyway and you don’t want to make their situation worse, or your own, so e-Visits definitely allow you to have that interaction safely at home, away from people.” (P3, Patient) “Our doctor has been on the telephone only. Until there’s a vaccine or something for the virus my personal opinion is that because of my husband’s lack of an immune system, I’m not going to risk taking him to a doctor so whatever way we have to deal with it is the way we will deal with it.” (P8, Caregiver) Interviewer: “Do you feel like these phone calls would be a good solution during this time to access health care?” Patient: “Yes, if you can’t get there, the phone call’s the next best thing” (P2, Caregiver).
In-person MDC is more preferable	“I prefer an in-person visit because then I can really talk to the person and everything. I had over a video, a meeting with the ALS team that are looking after me and it was very good but it was hard to explain some things to them and I had to show them my foot [audio cuts out] and it was hard for them to get a really good look at my foot and ankle, which is my main problem area right now” (P7, Patient) “Technology-wise it just, I get frustrated because nobody is seeing him. He can’t talk so he thinks of all these questions afterwards and writes them out. If he had the time to sit there and write them out in front of the doctor, then it wouldn’t take multiple calls. I’d rather do it face-to-face.” (P5, Caregiver) “As I say, you don’t get to know the people, I guess you don’t get to know your doctor intimately with these types of things but it is quite the same.” (P6, Patient)

In Canada, specialized MDCs are the standard of care in ALS management as they confer several care benefits, including the convenience of a single integrated clinic, increased use of adaptive equipment and treatment interventions, as well as improved quality of life and prolonged survival.^2,7^ Participants expressed travel to in-person MDCs was no longer viable because it was too exhausting or costly. In contrast, participants acknowledged that VC reduced fatigue because patients were able to rest immediately after long MDC sessions. VC also eliminated costs related to travel and parking. This benefit is particularly salient given the geography of Canada and the long distances rural and remote residents travel to receive in-person ALS multidisciplinary care, with specialized MDCs located in relatively few urban centers. Most of our participants drove at least 1 h (one-way) to attend their MDC (Table 1). VC can maintain continuity of care via remote monitoring of symptoms and provision of care without the need for travel.^8^

While the COVID-19 pandemic has presented unprecedented challenges for neuromuscular clinicians, providers have been able to make management recommendations by observing the condition of the patient by video conference.^3^ Patients in our study reported a high level of satisfaction with the virtual during the COVID-19 pandemic. All participants felt that virtual ALS MDCs allowed them to receive ongoing care in a safe manner. Participants reported less anxiety as they did not have to worry about unnecessary exposure to COVID-19 that could exacerbate their existing conditions (Table 1).

Notwithstanding the benefits of the virtual MDC, patients may prefer in-person care. In our study, half of the participants stated that they still preferred in-person MDCs due to a combination of technological problems, an impersonal feeling associated with virtual encounters, and the lack of physical examination. A successful virtual visit session relies on strong digital literacy, access to the Internet and technology, which may differ due to age, geography, education, and income.^9^ Multiple ALS-specific care endpoints require in-person assessments (e.g. pulmonary function tests). Also, the utility of virtual assessments in detecting relatively subtle/minor ALS disease progression and eliciting timely interventions is not validated. Therefore, while virtual MDCs may be an easier, less burdensome alternative to providing care, it may not always be preferable. This creates important implications for how to deliver patient-centered care as the convenience and cost-effective aspects of the virtual MDCs will remain even after the COVID-19 pandemic.

This pilot project adds to the body of literature exploring how VC could increase healthcare access for ALS patients.^3,8,10^ The results from our study are a starting point for further exploration to optimize ALS patient care. A mixed-methods approach incorporating quantitative methods (e.g. caregiver burden scores, patient-reported outcomes, clinical outcomes) would have further bolstered the study outcomes. As a patient’s ALS disease course progresses and as the pandemic resolves, there may be longitudinal changes in patients’ perceptions of VC. The cross-sectional nature of this study’s data is a limitation. The perceptions captured in this study do not necessarily correlate with adequate and timely ALS care delivery as recommended by the Canadian guidelines.^2^

Our findings indicate that virtual ALS MDC may alleviate some of the barriers to ALS care, but in-person clinics may be more preferable in certain contexts. Future studies should examine longitudinal changes in patient and caregiver perceptions of VC. There is also a need to consider formal integration of VC into ALS MDC pathways to enable accessible care to patients and families affected.
